# Multiple Introductions of Influenza A(H5N8) Virus into Poultry, Egypt, 2017

**DOI:** 10.3201/eid2405.171935

**Published:** 2018-05

**Authors:** Ahmed H. Salaheldin, Hatem Salah Abd El-Hamid, Ahmed R. Elbestawy, Jutta Veits, Hafez M. Hafez, Thomas C. Mettenleiter, Elsayed M. Abdelwhab

**Affiliations:** Alexandria University, Al Buhayrah, Egypt (A.H. Salaheldin);; Friedrich-Loeffler-Institut, Insel Riems-Greifswald, Germany (A.H. Salaheldin, J. Veits, T.C. Mettenleiter, E.M. Abdelwhab); Freie-Universität-Berlin, Berlin, Germany (A.H. Salaheldin, H.M. Hafez);; Damanhour University, Damanhour, Egypt (H.S. Abd El-Hamid, A.R. Elbestawy)

**Keywords:** H5N8, clade 2.3.4.4, highly pathogenic avian influenza virus, domestic birds, ducks, Egypt, Africa, migratory birds, viruses, influenza

## Abstract

After high mortality rates among commercial poultry were reported in Egypt in 2017, we genetically characterized 4 distinct influenza A(H5N8) viruses isolated from poultry. Full-genome analysis indicated separate introductions of H5N8 clade 2.3.4.4 reassortants from Europe and Asia into Egypt, which poses a serious threat for poultry and humans.

In Egypt, highly pathogenic avian influenza A(H5N1) clade 2.2.1 virus was introduced to poultry via migratory birds in late 2005 (*1*) and is now endemic among poultry in Egypt (*2*). Also in Egypt, the number of H5N1 infections in humans is the highest in the world, and low pathogenicity influenza A(H9N2) virus is widespread among poultry and has infected humans (*2*). Despite extensive vaccination, H5N1 and H9N2 viruses are co-circulating and frequently reported (*2*). In 2014, highly pathogenic avian influenza A(H5N8) virus clade 2.3.4.4 was isolated, mostly from wild birds, in several Eurasian countries and was transmitted to North America. However, in 2016 and 2017, an unprecedented epidemic was reported in Asia, Africa, and Europe (*3*). In Egypt, during November 30–December 8, 2016, a total of 3 H5N8 viruses were isolated from common coot (*Fulica atra*) (*4*) and green-winged teal (*Anas carolinensis*) (*5*). To provide data on the spread of the virus in poultry, we genetically characterized 4 distinct H5N8 viruses isolated from commercial poultry in Egypt in 2017.

During February–May 2017, a high mortality rate was observed for 48 poultry flocks in the Nile Delta, Egypt. Up to 20 tracheal and cloacal swab samples were collected from each flock for initial diagnosis by reverse transcription PCR and virus isolation at the Faculty of Veterinary Medicine, Damanhour University (Damanhour, Egypt). Results were positive for H5N8 virus in samples for 4 flocks not vaccinated for H5 in 3 governorates (Figure). Sudden deaths also occurred in 3 broiler chicken flocks (Ck12, Ck15, Ck21) and 1 duck flock (Dk18); mortality rates were 29%–52% (Technical Appendix 1 Table 1). No epidemiologic links between farms were observed. 

**Figure:**
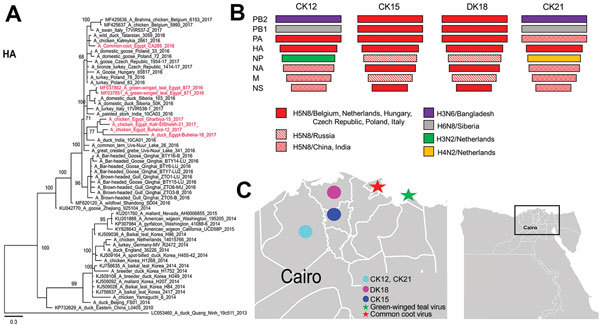
Characterization of highly pathogenic avian influenza A(H5N8) viruses of clade 2.3.4.4 from Egypt, 2017. A) Phylogenetic relatedness of the HA gene and schematic representation of potential precursors of different H5N8 viruses. The maximum-likelihood midpoint rooted tree was constructed by using MrBayes (http://mrbayes.sourceforge.net/). Red indicates viruses from this study. Scale bar indicates nucleotide substitutions per site. B) Putative ancestors of the different gene segments of H5N8 viruses from Egypt characterized in this study compared with reference viruses. C) Governorates in Egypt where H5N8 viruses had been reported in domestic birds (circles) and where viruses in birds had been previously reported (stars). Inset shows study location in Egypt. Ck, chicken farm; Dk, duck farm; HA, hemagglutinin; M, matrix; NA, neuraminidase; NP, nucleocapsid protein; NS, nonstructural; PA, polymerase acidic; PB, polymerase basic.

Positive samples were spotted onto FTA cards (*6*) and submitted to Friedrich-Loeffler-Institut (Insel Riems-Greifswald, Germany), where H5N8 virus was confirmed by reverse transcription PCR and full-genome sequences (*7*) from 4 viruses (GISAID [https://www.gisaid.org/] accession nos. EPI1104268–EPI1104299) (Technical Appendix 2). We retrieved sequences with high similarity and all H5N8 virus sequences from GISAID and GenBank and aligned them by Multiple Alignment using Fast Fourier Transform (https://mafft.cbrc.jp/alignment/server/index.html). The most highly related viruses are summarized in Technical Appendix 1 Table 2. We calculated sequence identity matrices in Geneious (https://www.geneious.com/) (Technical Appendix 1 Figure 1) and studied phylogenetic relatedness to H5N8 virus isolated in Eurasia and in Egypt by using IQtree (http://www.iqtree.org/). Representative viruses were selected for generation of maximum-likelihood midpoint rooted trees by MrBayes (http://mrbayes.sourceforge.net/) using a best-fit model (GTR+G) (*8*) and were further edited by using FigTree (http://tree.bio.ed.ac.uk/software/figtree/) and Inkscape (https://inkscape.org/en/). 

The hemagglutinin (HA) and neuraminidase (NA) genes of the 4 viruses shared 95.8%–99.2% nt and 93.1%–99.4% aa identity and shared 96.5%–99.2% nt and 94.2%–99.7% aa identity with viruses from wild birds in Egypt (*4*,*5*). Other segments showed 92.6%–99.6% nt and 96%–99.7% aa identity, where the polymerase acidic (PA) genes and proteins of viruses from Dk18 showed the lowest similarity to those of other viruses (Technical Appendix 1 Figure 1). 

All viruses possess the polybasic HA cleavage site PLREKRRKR/G and contain mammal-adaptation and virulence markers (*9*) in polymerase basic (PB) 2 (T63I, L89V, G309D, T339K, Q368R, H447Q, R477G), PB1 (A3V, L13P, K328N, S375N, H436Y, M677T), PA (A515T), HA (T156A, A263T; H5 numbering), matrix (M) 1 (N30D, T215A), and nonstructural (NS) 1 (P42S, T127N, V149A) proteins. Therefore, protection of humans and risk assessment of bird-to-human transmission is crucial. The NS1 protein from viruses from Ck15 and Ck18 is 217 aa long because of truncation in the C-terminus, whereas NS1 of the other H5N8 viruses from Egypt are 230 aa long. BLAST (http://blast.ncbi.nlm.nih.gov/Blast.cgi) analysis indicated that these 4 viruses differ from viruses isolated from birds in live bird markets in Egypt in 2016 (*4*,*5*). Gene segments were closely related to viruses isolated from wild birds, poultry, and zoo birds in Europe (including Belgium, Czech Republic, the Netherlands, Poland, Hungary), Russia, and Asia (including Bangladesh, China, India) (Figure; Technical Appendix 1 Figures 2, 3). 

HA of the 4 H5N8 viruses in this study clustered in 1 distinct branch (Figure), and NA clustered in 2 phylogroups (Technical Appendix 1 Figure 2). The PB2, nucleoprotein, M, and NS genes of viruses from Ck12 and Ck21 (from chickens in the same governorate, February and May 2017) clustered together, and the same genes from viruses from Dk18 and Ck15 (from ducks and chickens in 2 governorates) clustered in 2 distinct phylogenetic groups. However, viruses from Ck12 and Ck15 have similar but not identical PA gene segments (Technical Appendix 1 Figure 3). 

These data suggest 4 different introductions of H5N8 virus into poultry in Egypt, independent of viruses isolated from captive birds (*4*,*5*). Multiple separate introductions of H5N8 virus into Europe also occurred (*10*). Further studies are needed to identify the source(s) of introduction. The separate introductions of different reassortants of H5N8 clade 2.3.4.4 virus from Europe and Asia into Egypt indicate a serious threat for poultry and human health.

Technical Appendix 1 Additional information about influenza A(H5N8) viruses isolated from domestic poultry in Egypt, 2017.

Technical Appendix 2 Laboratories that submitted sequences to the GISAID database.
