# Supplementary material for: Multiple Introductions of Influenza A(H5N8) Virus into Poultry, Egypt, 2017
Source: Emerg Infect Dis. 2018 May;24(5):943–6. doi: 10.3201/eid2405.171935 (PMC5938773; doi:10.3201/eid2405.171935)
Supplement: Technical Appendix 1 — Additional information about influenza A(H5N8) viruses isolated from domestic poultry in Egypt, 2017. [file 17-1935-Techapp-s1.pdf]

Article DOI: https://doi.org/10.3201/eid2405.171935

Multiple Introductions of Influenza A(H5N8) Virus into Poultry, Egypt, 2017

Technical Appendix 1

Technical Appendix 1 Table 1. History of flocks infected with HPAIV H5N8 in 2017 characterized in this study

H5N8 Virus	Birds	Date	Governorate	No. birds	Age	Mortality
A/chicken/Egypt/Buheira-12/2017	Broilers	Mar.	Buheira	2000	29 days	35%
A/chicken/Egypt/Gharbiya/-15/2017	Broilers	Feb.	Gharbiya	1500	32 days	52%
A/duck/Egypt/Kafr Elshiekh-18/2017	Ducks	May	Kafr El-Sheikh	1500	32 days	29%
A/chicken/Egypt/Buheira-21/2017	Broilers	May	Buheira	2000	30 days	32%

Technical Appendix 1 Table 2. Identity (ID) of the Egyptian HPAIV H5N8 isolated in this study and related viruses with highest identity in the GenBank and GISAID databases

Segment	A/chicken/Egypt/Buheira-12/2017	ID%	A/chicken/Egypt/Gharbiya-15/2017	ID%	A/chicken/Egypt/ Kafr-Elshiekh-18/2017	ID%	A/duck/Egypt /Buheira-21/2017	ID%
PB2	A/duck/Bangladesh/26920/2015 (H3N6)	99.0	A/eurasian_wigeon/Netherlands/ 16015653-001/2016_(H5N8)	99.6	A/peacock/Belgium/1017/ 2017(H5N8)	98.9	A/duck/Bangladesh/26974/ 2015(H3N6)	98.9
PB1	A/domestic_duck/Siberia/ 49_feather/2016 (H6N8)	98.4	A/Eur_Wig/NL-Greonterp/16015653-001/2016 (H5N8)	99.6	A/M_Swan/NL- /16014462-019/2016 (H5N8)	99.7	A/domestic_duck/Siberia/ 49_feather/2016 (H6N8)	98.5
PA	A/Eur_Wig/NL-Greonterp/16015653-001/2016 (H5N8)	98.0	A/Eur_Wig/NL-Greonterp/16015653-001/2016 (H5N8)	99.3	A/Cygnus olor/Belgium/1567/201 7(H5N8)	98.6	A/Bar-headed Goose/Qinghai/BTY9-LU/2016 (H5N8)	98.7
HA	A/mute swan/Czech Republic/1296-17_1/2017 (H5N8)	98.9	A/Mallard/Hungary/1574a/2017 (H5N8)	99.1	A/turkey/Poland/83/2016 (H5N8)	98.9	A/mute swan/Czech Republic/1296-17_1/2017 (H5N8)	97.0
NP	A/mallard duck/Netherlands/ 16/2012(H3N2)	97.6	A/mute swan/Kaliningrad/132/2017 (H5N8)	99.7	A/gadwall/Kurgan/2442/ 2016 (A/H5N8)	98.3	(A/mallard duck/Netherlands/18/2012 (H4N2))	97.9
NA	A/Brown-headed Gull/Qinghai/ZTO5-K/2016 (H5N8)	99.1	A/Eur_Wig/NL- /16015653-001/2016 (H5N8)	99.4	A/peacock/Belgium/1017/ 2017(H5N8)	97.3	A/Brown-headed Gull/Qinghai/ZTO6-MU/2016 (H5N8)	98.7
M	A/duck/India/10CA01/2016 (H5N8)	99.7	A/gadwall/Kurgan/2442/2016 (H5N8)	99.2	A/gadwall/Kurgan/2442/ 2016 (A/H5N8)	99.5	A/duck/India/10CA01/2016 (A/H5N8)	99.6
NS	A/duck/India/10CA01/2016 (A/H5N8)	99.3	A/Mallard/Hungary/1574a/2017 (A/H5N8)	99.9	A/goose/Italy/17VIR6358-3/2017 (A/H5N8)	99.4	A/duck/India/10CA01/2016 (A/H5N8)	99.1

HA	Ck12	Ck15	Dk18	CK21	Coot	Teal877	Teal871
Ck12	ID	99.1	99	97.4	98.5	98.5	98.4
Ck15	99.4	ID	99.2	97.1	98.7	98.5	98.4
Dk18	98.7	98.9	ID	97	98.8	98.3	98.2
CK21	97.8	97.7	97	ID	96.7	96.5	96.5
Coot	99.2	99.4	99.1	97.5	ID	99.1	99.1
Teal877	99.6	99.4	98.7	97.8	99.6	ID	99.9
Teal871	99.4	99.2	98.5	97.7	99.4	99.8	ID

PA	Ck12	Ck15	Dk18	CK21	Teal877	Teal871
Ck12	ID	98.1	93.6	94.2	94.3	94.3
Ck15	99.1	ID	92.6	93.2	94.3	94.3
Dk18	96.0	96.3	ID	97.7	94.5	94.5
CK21	97.0	97.3	97.2	ID	95.1	95.1
Teal877	98.0	98.3	96.3	97.6	ID	100.0
Teal871	98.0	98.3	96.3	97.6	100.0	ID

NA	Ck12	Ck15	Dk18	CK21	Coot	Teal877	Teal871
Ck12	ID	98	96.3	98.8	98.7	98.7	98.7
Ck15	97.8	ID	96.6	97.7	99.2	98.5	98.5
Dk18	93.8	95.1	ID	95.8	97.1	96.6	96.6
CK21	97.8	97.2	93.1	ID	98.5	98.3	98.4
Coot	98.0	99.7	95.3	97.4	ID	99.2	99.3
Teal877	97.6	98.7	94.2	96.5	98.9	ID	99.9
Teal871	97.8	98.9	94.4	96.8	99.1	99.7	ID

NP	Ck12	Ck15	Dk18	CK21	Teal877	Teal871
Ck12	ID	97.1	96.5	98.0	94.0	93.9
Ck15	96.9	ID	98.5	97.5	95.2	95.1
Dk18	96.5	99.5	ID	97.0	94.8	94.7
CK21	96.7	98.9	98.5	ID	94.6	94.5
Teal877	96.3	99.3	98.9	98.3	ID	99.9
Teal871	96.1	99.1	98.7	98.1	99.7	ID

PB2	Ck12	Ck15	Dk18	CK21	Teal877	Teal871
Ck12	ID	98.4	98.0	99.3	97.2	97.1
Ck15	98.6	ID	98.6	98.5	97.0	96.9
Dk18	98.4	98.6	ID	98.2	96.9	96.8
CK21	99.0	98.8	98.5	ID	97.1	97.0
Teal877	98.4	98.4	98.1	98.2	ID	99.9
Teal871	98.2	98.2	98.0	98.1	99.8	ID

M1	Ck12	Ck15	Dk18	CK21	Teal877	Teal871
Ck12	ID	98.1	98.8	99.6	99.4	99.4
Ck15	98.4	ID	98.5	98.2	98.1	98.1
Dk18	98.4	98.4	ID	98.6	98.5	98.5
CK21	99.6	98.8	98.8	ID	99.3	99.3
Teal877	99.6	98.0	98.0	99.2	ID	100.0
Teal871	99.6	98.0	98.0	99.2	100.0	ID

PB1	Ck12	Ck15	Dk18	CK21	Teal877	Teal871
Ck12	ID	95.0	95.1	99.5	95.1	95.2
Ck15	98.5	ID	99.4	95.1	98.7	99.0
Dk18	98.8	99.7	ID	95.2	98.9	99.2
CK21	99.4	98.8	99.0	ID	95.2	95.3
Teal877	98.0	98.4	98.6	98.2	ID	99.3
Teal871	98.6	99.0	99.3	98.9	98.8	ID

NS1	Ck12	Ck15	Dk18	CK21	Teal877	Teal871
Ck12	ID	98.6	97.7	99.5	99.1	99.1
Ck15	98.6	ID	99.1	98.2	98.6	98.6
Dk18	97.7	99.1	ID	97.3	97.7	97.7
CK21	99.5	98.2	97.3	ID	98.6	98.6
Teal877	99.1	98.6	97.7	98.6	ID	100
Teal871	99.1	98.6	97.7	98.6	100	ID

Technical Appendix 1 Figure 1. Genetic identity of different genes and proteins of HPAIV H5N8 clade 2.3.4.4 isolated from domestic poultry in Egypt in 2017. Given are the nucleotide and amino acid identity matrices ($^{nucleotide}ID_{amino\ acid}$) of H5N8 isolated from poultry in this study compared to the wild bird viruses. ID= identical, Ck12= A/chicken/Egypt/Buheira-12/2017, Ck15= A/chicken/Egypt/Gharbiya-15/2017, Dk18= A/chicken/Egypt/Kafr-Elshiekh-18/2017, Ck21= A/duck/Egypt/Buheira-21/2017, Coot= A/Common coot/Egypt/CA285/2016, Teal 877= A/green winged teal/Egypt/877/2016, Teal 871= A/green winged teal/Egypt/871/2016. Only HA and NA gene sequences are available for the common coot virus. Sequences were aligned and identity matrices were calculated using Geneious version 10.

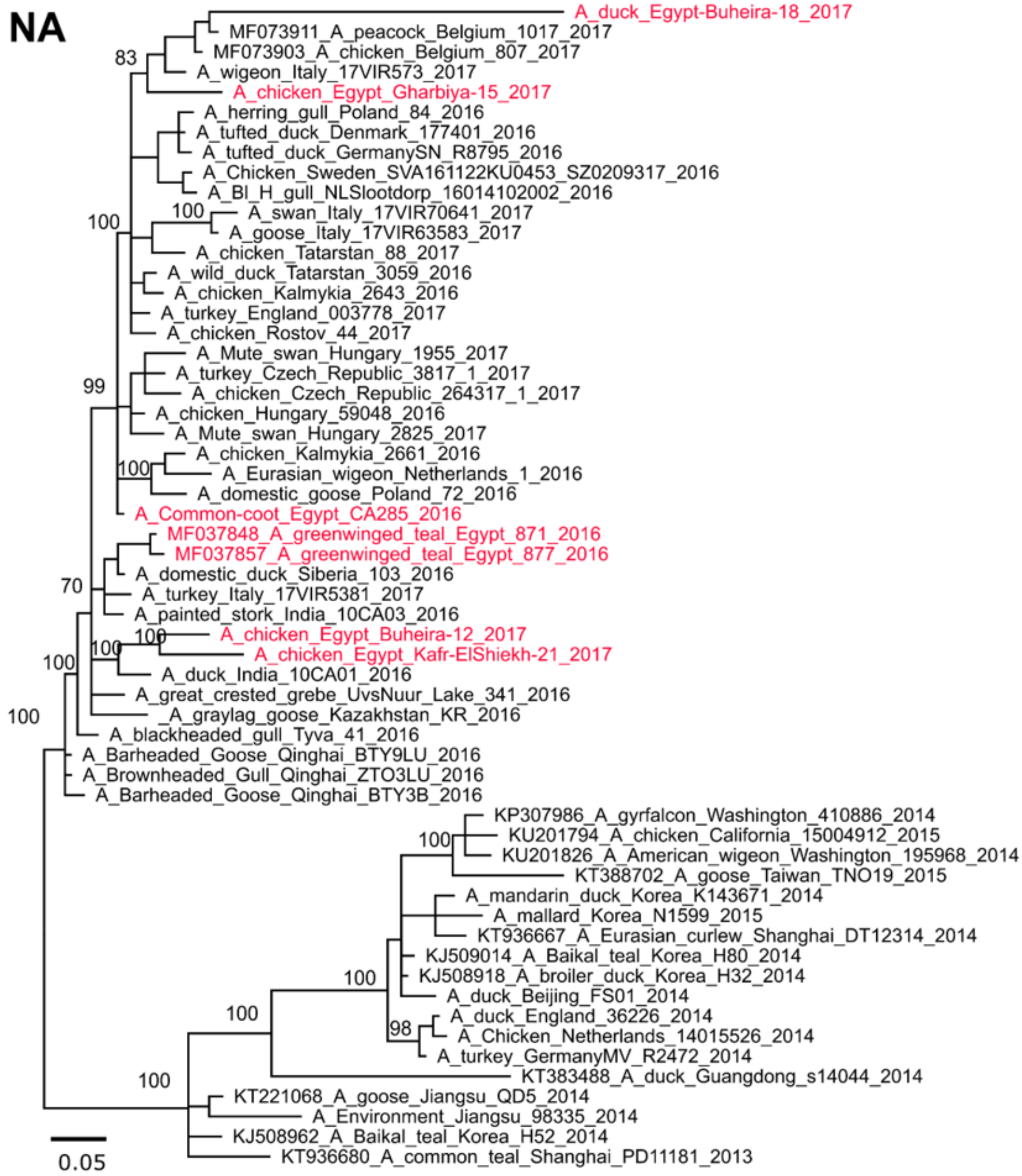

Technical Appendix 1 Figure 2. Phylogenetic relatedness of the NA gene segments of the H5N8 2.3.4.4 viruses from domestic birds in Egypt in 2017. All available sequences of NA of HPAIV H5N8 were retrieved from the GISAID and GenBank databases and aligned using MAFFT. Trees were generated by IQtree and representative viruses were selected for further analysis. Maximum Likelihood mid-point rooted trees were constructed by MrBayes implementing 4 runs, 1000.000 replicates and 10% burn-in using best fit model GTR+G according to BIC value. Egyptian viruses are shown in red.

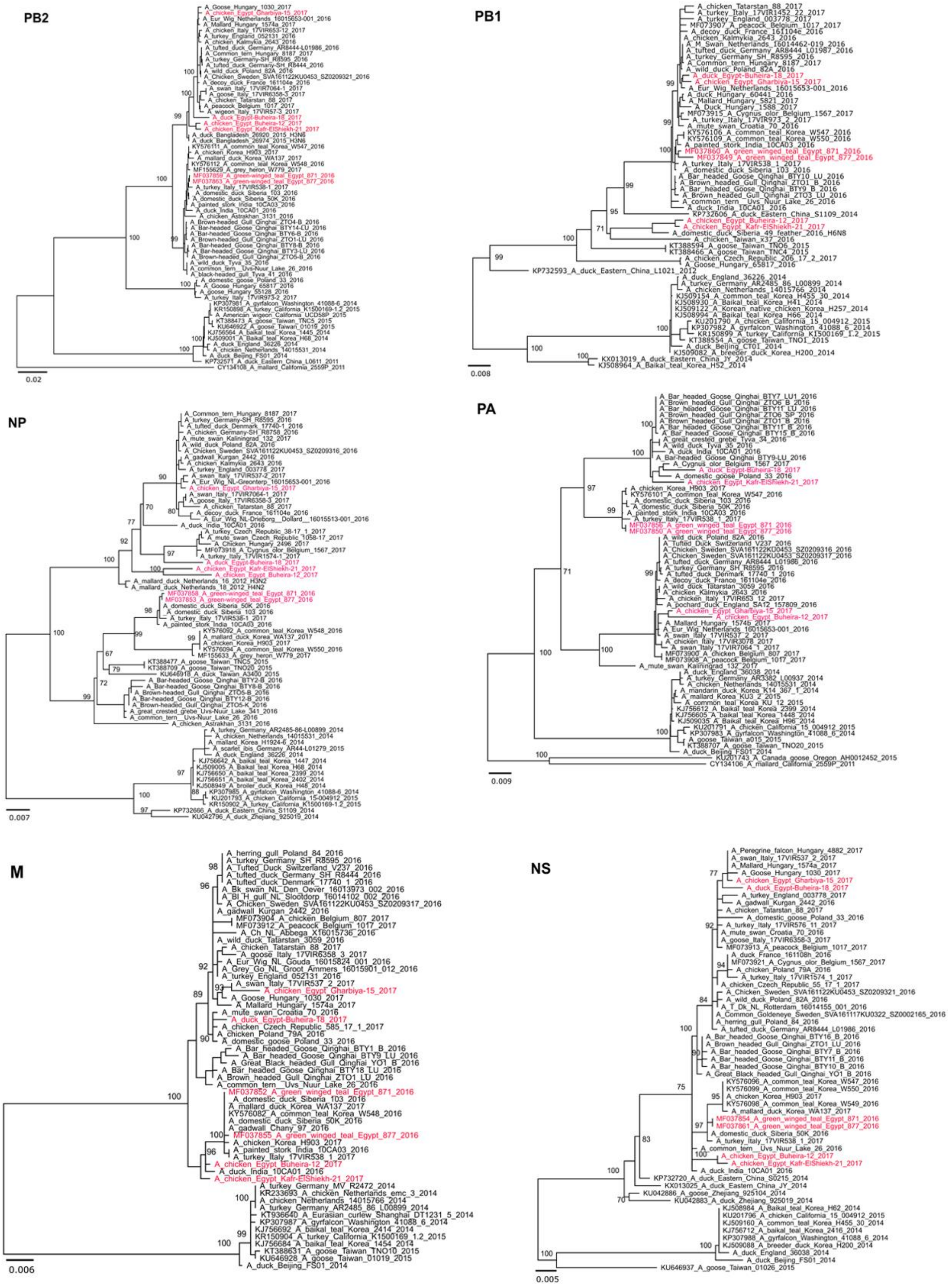
PB2
0.02
PB1
0.008
NP
0.007
PA
0.009
M
0.006
NS
0.005

Technical Appendix 1 Figure 3. Phylogenetic relatedness of non-HA/NA gene segments of the H5N8 2.3.4.4 viruses from domestic birds in Egypt in 2017. All available sequences of H5N8 viruses were retrieved from the GISAID and GenBank databases. First tree was generated by IQtree and representative viruses were selected for further analysis. Maximum Likelihood mid-point rooted trees were generated by MrBayes using 4 runs each of 1000.000 replicates and 10% burn-in after selection of the best fit model GTR+G according to BIC value as implemented in Topali v2. All trees were edited and prepared for publishing using FigTree and Inkscape.
